# Bidirectional effects of physical activity and sleep on health: evidence and future directions

**DOI:** 10.3389/fspor.2026.1739588

**Published:** 2026-02-20

**Authors:** Vicente Javier Clemente-Suárez, Laura Redondo-Flórez, Ana Isabel Beltrán-Velasco, Domingo Jesús Ramos-Campo, Pablo Ruisoto, Rodrigo Yáñez-Sepúlveda, Alexandra Martín-Rodríguez, José Francisco Tornero-Aguilera

**Affiliations:** 1 Faculty of Medicine, Health and Sports, Universidad Europea de Madrid, Villaviciosa de Odón, Madrid, Spain; 2 Grupo de Investigación en Cultura, Educación y Sociedad, Universidad de la Costa, Barranquilla, Colombia; 3 Department of Biomedicine and Dentistry, Faculty of Biomedical Sciences and Sports, Universidad Europea de Andalucía, Málaga, Spain; 4 Psychology Department, Facultad de Ciencias de la Vida y la Naturaleza, Universidad Antonio de Nebrija, Madrid, Spain; 5 LFE Research Group, Department of Health and Human Performance, Faculty of Physical Activity and Sport Science-INEF, Universidad Politécnica de Madrid, Madrid, Spain; 6 Department of Health Sciences, Public University of Navarre, Pamplona, Spain; 7 Faculty of Education and Social Sciences, Universidad Andres Bello, Viña del Mar, Chile; 8 School of Medicine, Universidad Espíritu Santo, Samborondón, Ecuador; 9 Faculty of Education Sciences, UNIE Universidad, Madrid, Spain; 10 Department of Sport Sciences, Faculty of Sport and Health Sciences, Fit Generation Research Institute, Andorra la Vella, Andorra

**Keywords:** physical activity, physical activity performance, sleep, sleep duration, sleep quality

## Abstract

**Aim:**

Sleep and physical activity are two important lifestyle factors that significantly influence overall health and wellbeing. This comprehensive review aims to provide a detailed understanding of the interplay between sleep and physical activity habits.

**Methods:**

A narrative review was conducted through a comprehensive assessment of primary and secondary sources, incorporating scientific publications from databases such as MedLine, Cochrane, Embase, PsychINFO, and Cinahl. The inclusion criteria focused on studies published between 2000 and 2025, addressing topics such as physical activity, sleep quality, sleep disorders, energy balance, and related health outcomes. Exclusion criteria included gray literature, unpublished studies, books, conference proceedings, and dissertations.

**Results:**

The results highlight the complex bidirectional relationship between sleep and physical activity. Regular physical activity improves sleep quality and duration, while adequate sleep enhances physical activity performance and recovery. Sleep disorders negatively affect physical activity engagement, but interventions involving exercise demonstrate significant potential in mitigating these effects.

**Conclusions:**

In conclusion, understanding the multifaceted interactions among sleep, physical activity, and nutrition is crucial for promoting overall health and wellbeing. Future research should leverage advancements in wearable technology, personalized interventions, and precision medicine approaches to optimize these interrelated behaviors and their health impacts.

## 1 Introduction

Sleep and physical activity are fundamental lifestyle factors that significantly influence overall health and wellbeing (1). Understanding the interplay between sleep and physical activity habits is crucial for developing effective strategies to enhance health outcomes. Numerous studies have explored the complex relationship between these behaviors, highlighting their bidirectional nature and the intricate mechanisms underlying their interactions. Research has shown that physical activity can have a substantial impact on sleep quality and duration. Regular physical activity has been associated with improved sleep outcomes, including enhanced sleep efficiency (SE), reduced sleep latency, and increased total sleep time (2, 3). In addition, higher levels of physical activity have been linked to a decreased risk of developing sleep disorders such as insomnia (4). However, the timing, intensity, and duration of physical activity can influence sleep, with vigorous exercise close to bedtime potentially disrupting sleep (5).

Conversely, sleep plays a crucial role in physical activity performance and recovery. Adequate sleep duration and quality have been associated with better athletic performance, cognitive function, and motor skills (6, 7). Insufficient sleep, on the other hand, can lead to decreased exercise capacity, impaired reaction time, and increased injury risk (8, 9). Moreover, sleep has been shown to affect the physiological processes involved in postexercise recovery, including muscle repair and glycogen restoration (10). Furthermore, the relationship between sleep and physical activity extends to the regulation of energy balance and weight management. Sleep deprivation (SD) has been associated with alterations in appetite-regulating hormones, such as increased ghrelin levels and decreased leptin levels, leading to increased food intake and a higher risk of obesity (11). Physical activity can modulate these hormonal profiles, potentially mitigating the negative effects of sleep deprivation on energy balance and weight control (12).

Importantly, sleep disorders can significantly impact physical activity engagement and performance. Conditions such as obstructive sleep apnea have been associated with reduced physical activity levels and exercise capacity (13). Conversely, physical activity interventions, including exercise training and physical therapy, have shown promise in improving sleep quality and reducing the risk of sleep disorders (14, 15). The complex relationship between sleep and physical activity involves multiple underlying mechanisms. Circadian rhythms, hormonal regulation, and neural pathways play key roles in coordinating the interactions between these behaviors (16, 17). Moreover, nutrition has emerged as a significant factor that can influence both sleep and physical activity outcomes (18). Optimal dietary patterns and nutrient intake can support healthy sleep and physical activity, while inadequate nutrition can impair performance in both domains.

To comprehensively understand the interplay between sleep and physical activity habits, this review synthesizes existing knowledge across a variety of research domains. We will explore the impact of physical activity on sleep quality and duration, the effects of sleep on physical activity performance and recovery, and the role of sleep in energy balance and weight management. In addition, we will investigate the influence of sleep disorders on physical activity engagement and the potential benefits of physical activity interventions for improving sleep outcomes. Moreover, we will examine the impact of timing, intensity, and duration of physical activity on sleep, and the reciprocal effects of sleep duration and quality on physical activity. Furthermore, we will delve into the potential underlying mechanisms, including circadian rhythms, hormonal regulation, and neural pathways. The interrelationships between nutrition, sleep, and physical activity will also be explored, along with potential synergies for intervention strategies. Lastly, we will highlight future perspectives, such as advancements in wearable technology, personalized interventions, and precision medicine approaches, to further enhance our understanding and optimize the interplay between sleep and physical activity habits.

Narrative reviews are essential for academic knowledge as they provide a comprehensive understanding of a topic, integrate diverse evidence sources, stimulate critical analysis, identify research gaps, and guide future investigations. Unlike systematic reviews, narrative reviews capture the breadth and depth of a subject, serving as valuable educational tools and facilitating knowledge acquisition. They excel at synthesizing qualitative and quantitative research, fostering discussions, generating hypotheses, and proposing theoretical models. Moreover, narrative reviews adapt to evolving research, ensuring relevance and contributing to the advancement of knowledge across various academic disciplines.

To achieve the objectives of this narrative review, a comprehensive assessment was conducted, incorporating primary sources, such as scientific publications, and secondary sources, such as bibliographic indexes, web pages, and databases. The primary focus of this narrative review was to gain a thorough understanding of the intricate relationship between sleep and physical activity habits. The search criteria encompassed English manuscripts published between 2000 and 2025, except for classic literature, while excluding gray literature. Furthermore, additional references were sought through retrieved articles, practice guidelines, editorials, and letters. To encompass the multifaceted nature of mental health, a range of databases including MedLine, Cochrane, Embase, PsychINFO, and Cinahl were utilized. Studies addressing topics related to nutrition, physical activity, sleep, sleep quality, sleep disorders, physical performance, recovery, activity habits, medicine, and wearable technology were included. Exclusion criteria consisted of research falling outside the specified time, topics beyond the scope of this review, and unpublished studies, books, conference proceedings, abstracts, and dissertations. We extracted the information and allocated the data based on our respective areas of expertise.

## 2 Physical activity as a modulator of sleep: effects, mechanisms, and exercise prescription

Sleep is a fundamental component of physical and mental health, with current recommendations suggesting a minimum of 7 h of sleep per night for adults to support optimal functioning. In recent years, growing evidence has highlighted physical activity as a key behavioral factor capable of modulating sleep quality and, to a lesser extent, sleep duration, reinforcing the bidirectional relationship between these two lifestyle behaviors (19).

### 2.1 Effects of physical activity on sleep quality and duration

A substantial body of evidence supports the beneficial effects of physical activity on sleep, particularly on subjective sleep quality. A meta-analysis demonstrated that exercise interventions consistently improve sleep quality across diverse populations. Acute exercise has been associated with modest improvements in sleep onset latency (SOL), sleep efficiency, slow-wave sleep (SWS), and wake time after sleep onset, whereas chronic exercise appears to exert small-to-moderate benefits on sleep quality and more limited effects on total sleep time (20, 21).

Importantly, accumulating evidence suggests that physical activity is more strongly and consistently associated with improvements in sleep quality than sleep duration. While sleep duration and quality are conceptually related, they appear to be partially independent constructs, and improving sleep quality may represent a more achievable and clinically relevant target for exercise-based interventions.

### 2.2 Underlying mechanisms linking exercise and sleep

The sleep-promoting effects of physical activity are mediated through multiple, interacting physiological mechanisms. Exercise influences hypothalamic–pituitary–adrenal axis regulation, autonomic balance, circadian rhythmicity, and neuroendocrine signaling, all of which play critical roles in sleep regulation (22).

Regular physical activity contributes to improved circadian alignment and sleep–wake regularity, potentially reducing stress-related hyperarousal and attenuating cortisol dysregulation associated with sleep disruption. In addition, exercise-induced increases in neurotrophic factors such as brain-derived neurotrophic factor and insulin-like growth factor have been linked to enhanced neuroplasticity, reduced inflammation, and improved cognitive and emotional regulation, which may indirectly support better sleep quality (23, 24).

Other proposed mechanisms include exercise-related modulation of thermoregulation and inflammatory signaling. Moderate elevations in body temperature following exercise may facilitate subsequent nocturnal temperature downregulation, promoting sleep onset and slow-wave sleep. Similarly, exercise-induced changes in inflammatory cytokines appear to follow a dose-dependent pattern, whereby moderate activity supports restorative sleep, whereas excessive exercise loads may disrupt sleep continuity. Overall, no single mechanism fully explains the sleep-enhancing effects of exercise, highlighting the multifactorial nature of this relationship.

### 2.3 Physical activity as a non-pharmacological intervention for sleep disorders

Beyond general sleep enhancement, physical activity has emerged as a promising non-pharmacological strategy for improving sleep disturbances and reducing the burden of sleep disorders. Insomnia and obstructive sleep apnea (OSA) are among the most prevalent and costly sleep disorders, affecting a substantial proportion of the adult population (25). Exercise interventions have demonstrated beneficial effects on sleep quality, daytime functioning, and cardiometabolic health in individuals with sleep disorders, reinforcing their role as an adjunctive or alternative treatment option.

In particular, moderate-intensity exercise has been associated with improvements in sleep architecture and symptom severity in individuals with obesity-related OSA, potentially through reductions in body mass index, improvements in respiratory function, and enhanced upper airway muscle tone. Evidence regarding restless legs syndrome remains more limited, but emerging intervention studies suggest that regular, moderate exercise may reduce symptom severity and improve sleep quality in affected individuals (25).

### 2.4 Exercise prescription: timing, intensity, and dose

While physical activity generally exerts beneficial effects on sleep, the timing, intensity, and volume of exercise appear to modulate these outcomes. Observational and experimental studies indicate that moderate-intensity exercise is consistently associated with improved sleep quality, whereas high-intensity exercise performed close to bedtime may delay sleep onset and reduce sleep efficiency in some individuals. However, recent systematic reviews suggest that evening exercise does not universally impair sleep, particularly when completed sufficiently before bedtime and tailored to individual tolerance and chronotype.

Exercise intensity appears to play a more critical role than time of day, with moderate-intensity sessions generally yielding the most favorable sleep outcomes. Current physical activity guidelines—recommending at least 150 min per week of moderate-intensity or 75 min per week of vigorous-intensity exercise—are associated with small-to-moderate improvements in subjective sleep quality (26), with greater benefits observed when exercise volume or intensity slightly exceeds minimum recommendations (27, 28).

Nevertheless, excessive exercise duration or volume may surpass an individual threshold and negatively affect sleep, underscoring the importance of personalized exercise prescription. Aerobic, resistance, and mind–body activities such as walking, yoga, tai chi, and Pilates have all demonstrated sleep-enhancing effects, allowing flexibility in intervention design based on individual preferences and clinical context (29).

### 2.5 Strength of evidence, inconsistencies, and research gaps

Overall, the evidence supporting a positive association between physical activity and sleep quality is relatively robust, particularly for subjective sleep outcomes and moderate-intensity exercise interventions in adult populations. Meta-analyses and randomized controlled trials consistently indicate improvements in sleep quality, sleep onset latency, and sleep efficiency following regular physical activity, supporting a clinically meaningful relationship. In contrast, evidence linking physical activity to changes in sleep duration is less consistent, with generally small or null effects reported across studies (23, 28, 30).

Despite these strengths, several inconsistencies remain in the literature. Findings related to exercise timing, intensity, and volume are heterogeneous, with some studies reporting sleep-disruptive effects of late or high-intensity exercise, while others observe neutral or even beneficial outcomes. These discrepancies are likely influenced by methodological differences, individual factors such as chronotype and fitness level, and variations in sleep assessment methods, limiting direct comparability across studies (31, 32).

Important gaps also persist regarding causality and population-specific effects. Much of the existing evidence is observational, precluding definitive conclusions about causal pathways between physical activity and sleep. Moreover, most intervention studies have been conducted on healthy adults, with comparatively limited data in older adults, adolescents, clinical populations, and individuals with sleep disorders. Sex-specific responses, long-term adherence, and interindividual variability in responsiveness to exercise-based sleep interventions remain underexplored (33, 34). Although emerging evidence suggests sex-specific responses to exercise–sleep interactions, with aerobic exercise appearing particularly effective for improving sleep quality in women, the role of hormonal status and long-term sleep recovery remains insufficiently explored. Moreover, while short-term exercise interventions (8–12 weeks) reliably improve sleep outcomes, maintaining long-term adherence poses a major challenge, with benefits often diminishing over time in the absence of continued support. Finally, interindividual variability in sleep responses to exercise remains poorly understood, as recent work suggests that natural within-subject fluctuations may exceed true physiological responsiveness, underscoring the need for more rigorous, long-term and personalized intervention studies (35, 36).

Finally, there is a need for greater consistency and rigor in intervention design. Future studies should adopt standardized, objective measures of both physical activity and sleep, incorporate sufficiently long follow-up periods, and explicitly account for moderating factors such as baseline sleep status, chronotype, and comorbidities. Addressing these gaps will be essential to advancing personalized, evidence-based exercise prescriptions aimed at optimizing sleep health.

## 3 The effects of sleep on physical activity performance and recovery

Numerous studies have shown that adequate sleep duration and quality are closely linked to improved physical activity development. Hence, adequate rest allows the body to recover and repair itself, leading to enhanced physical performance and skill acquisition. During sleep, the brain consolidates memories and motor skills acquired during physical activity. In turn, sufficient sleep facilitates the encoding and integration of new movement patterns, enhancing motor learning and coordination (37, 38). Moreover, sleep plays a critical role in hormonal regulation, including the secretion of growth hormone (GH), which is essential for muscle growth and tissue repair. Adequate sleep promotes optimal hormone balance, facilitating the development of lean muscle mass and overall physical performance (39).

Several authors have highlighted the detrimental impact of sleep loss on exercise performance. Sleep loss has been shown to exert a harmful effect on individuals, with numerous adverse consequences documented due to sleep deprivation. Thus, sleep deprivation involves a reduction in muscular strength and endurance (40, 41), a modification in emotional mood, including decreased motivation levels and increased perceived effort (42–44), and alterations in cognitive processing developments, such as diminished fine motor skills (45). Other authors have described the negative impact of sleep deprivation on strength. In a study conducted of judokas, partial sleep deprivation timed at the end of the night decreased muscle strength and power during short-term maximal performances (46). It has also been described that sleep deprivation may have an adverse impact on endurance. Recent research has shown that total sleep deprivation in cyclists can impair prolonged self-paced endurance performance by approximately 10% for a duration of around 60 min (47). This highlights the significant impact sleep deprivation can have on an individual's ability to sustain endurance activities at a high level. Similarly, in a study conducted in military populations, it was observed that partial sleep deprivation can increase both cardiorespiratory and psychological strain, ultimately limiting an individual's capacity for high-intensity endurance activities (48). Sleep disruption has also been negatively related to a reduction in aerobic power. In one study, athletes completed cycling tests under different sleep conditions, including normal sleep, partially deprivation (4 h), and complete sleep deprivation. Aerobic performance was impaired by 11.4% under complete deprivation and 4.1% under partially deprivation (49). Sleep deprivation negative impacts tasks requiring a high level of precision. For example, in a study of dart-throwing athletes, sleep deprivation was associated with diminished alertness, increased fatigue, and small but measurable declines in performance (45).

On the topic of physical recovery, several researchers have pointed out how sleep loss could have a negative impact. Optimal recovery is essential for repairing damaged tissues, replenishing energy stores, and adapting to the physiological demands of exercise. Sleep deprivation disrupts these recovery processes, compromising the body's ability to repair and restore itself after physical activity. These findings may be explained by the fact that sleep disruption may compromise GH release, which has been extensively related to sleep regulation (50). During sleep, the body releases GH, which plays a crucial role in tissue repair and muscle growth. However, inadequate sleep can disrupt the normal secretion of GH, impairing the body's ability to recover effectively. Recent literature has emphasized the key role that GH plays in regulating protein anabolism (51). This mechanism is mediated through IGF1-dependent endocrine and paracrine processes, as well as IGF1-independent pathways. The overall impact of GH on whole-body protein metabolism involves the redirection of amino acids toward synthesis, while reducing their irreversible oxidative loss. However, it is important to note that the effects of GH on different tissues vary, with distinct impacts on muscle tissue compared to extra-muscular tissues. In addition to its effects at the protein level, GH also involves metabolic processes, which promote energy production in muscle cells through different biochemical sources. Hence, GH has been described as enhancing glycogenolysis and gluconeogenesis as well as stimulating lipid oxidation and diminishing carbohydrate utilization (52, 53). Moreover, apart from GH activity, sleep deprivation can also impact glycogen replenishment, which is vital for restoring energy stores in the muscles and liver. Hence, glycogen serves as a readily available source of glucose when the body requires energy. In one study, 10 athletes underwent 30 h of sleep deprivation during consecutive-day intermittent sprint performances, after which their muscle glycogen content was examined. The study involved a single-day “baseline” session, followed by two consecutive-day experimental trials. These trials were separated by either a normal night's sleep or a period of no sleep. As a result, individuals who were sleep deprived had significantly reduced muscle glycogen rates compared to those who had sufficient sleep (54). This decrease in glycogen replenishment can result in decreased energy availability during subsequent exercise sessions, hindering overall performance and recovery. These results highlight the importance of adequate quality and duration for restoring body functioning and supporting recovery after exercise. Moreover, incorporating daytime napping has been suggested as a beneficial approach to enhance recovery, particularly in intensive training regimens that involve multiple training sessions within a single day (55).

## 4 The role of sleep in regulating energy balance and weight management

The biological mechanisms that regulate sleep are determined by the genetic information encoded by the sensory marker light. Within this framework, essential physiological processes that facilitate the production of sleep can be differentiated and are activated at different intensities throughout the 24-hour light-dark cycle (56, 57). The purpose of these processes is to maintain the internal balance of the sleep–wake periods (58).

Different hormones and cellular assemblies involved in sleep-related processes have been identified. Melatonin, which is produced in the pituitary gland and synthesized from tryptophan, is released into the bloodstream, and reaches its highest levels during the night (59). This hormone acts mainly on the suprachiasmatic nucleus (SCN), which maintains neuronal circuits connected to the retina, responsible for detecting sunlight (60).

Conversely, adenosine facilitates reactive homeostasis via cellular metabolic processes linked to neural activity (61). Adenosine is an endogenous purine nucleoside and has a negative dromotropic effect (62). It accumulates in the lateral hypothalamus and can inhibit information access by binding to receptors in the ventrolateral preoptic region, allowing the arrest of ascending reticular system activity (63).

In addition, another factor regulating sleep is body temperature. At the end of the day, daily activity decreases, accompanied by a reduction in body temperature. This allows one to enter a state of rest, characterized by a decrease in neural activity and the induction of sleep. Body temperature usually decreases at the onset of sleep and during the second stage of the full cycle, when it reaches its lowest levels (64, 65).

During sleep, there is secretion of different hormones such as the thyroid-stimulating hormone secreted in the pituitary gland, the GH secreted in the hypothalamus, and cortisol secreted in the adrenal glands (66). These hormones contribute to maintaining physiological homeostasis in living beings and are essential for hormonal stabilization (67, 68).

Endocrine homeostasis is closely related to different neurological processes of brain repair and restoration of executive functions (69). For example, during deep sleep, the consolidation of learning and memory is established, particularly during rapid-eye movement (REM) phases (70). Sleep also plays a fundamental role in the induction and regulation of humoral and cellular immunity (71). Cellular immunity is activated through tumor necrosis factor (TNF) and interferon, which drive cells determined for phagocytosis of harmful particles or viruses. Specialized cells, such as natural killer and monocytes, produce IL4, IL6, IL10, and IL13 during sleep. Sleep cycle disturbances can impair immune modulation and the ability to deal with harmful or dangerous cells (72).

Energy balance is maintained through several processes, which can be genetic and environmental, including stress, diet, physical activity, microbiota, and sleep (73, 74). However, all of them are activated through the genes MC4R and FTO. MC4R, a G protein-coupled receptor, regulates appetite through the hypothalamic leptin–melanocortin pathway. FTO encodes the enzyme FTO, a member of the Fe (II) family and 2-oxoglutarate-dependent oxygenase. This is involved in different functions, such as the demethylation of nucleic acids *in vitro* (75–77).

Sleep determines the release of hormones that have intracellular reparative capacity, as well as those hormones that are involved in the regulation of energy balance and weight control (78, 79). When sleep disturbances occur, the efficiency of the neuroendocrine system is compromised, as in the case of sleep apnea. This pathology is dangerous because normal air exchange is interrupted, leading to high cardiac excitation, high levels of stress, and shortness of breath (80, 81).

For all these reasons, there is growing interest in the relationship between sleep and eating patterns, energy balance, and body weight. Recent studies have demonstrated a direct relationship between adequate sleep patterns and metabolic alterations, which otherwise contribute to pathologies such as obesity, metabolic syndrome, or intestinal alterations (82). Studies have shown that the higher the quality of sleep, the greater the chances of reducing body mass index. Similarly, the lower the quantity and quality of sleep during the first few years of life, the higher the risk of obesity in childhood and adulthood (83, 84). Other studies using physiological sleep measures such as actigraphy or polysomnography (PSG), which provide records motor activity, have reported data on sleep deprivation and its direct association with increased risk of overweight and obesity (85, 86).

In animal models, previous studies have shown that sleep modulation is altered during the development of obesity, suggesting that the regulatory mechanisms of sleep and body weight may be interconnected (87). Sleep deprivation has been linked to alterations in hypothalamic peptide signaling, particularly within the orexin neuropeptide system, in the lateral and posterior hypothalamus (88). In addition, after a few days of sleep deprivation, the plasma concentration of leptin is reduced while increasing ghrelin levels, a hormone involved in the regulation of appetite and nutritional balance. Ghrelin is also involved in other physiological processes such as insulin secretion (89, 90). In studies in which people were maintained on a 4-h daily sleep schedule, a significant decrease in glucose tolerance was observed, as well as increases in plasma insulin concentration and insulin-like growth factor or IGF-1, a hormone that modulates growth hormone effects (91). Moreover, sleep deprivation affects the proper functioning of glucose homeostasis, and altered sleep patterns are implicated in insulin sensitivity and glucose tolerance. Selective sleep deprivation in the third stage, or low non–rapid eye movement sleep (SNREM), decreases insulin sensitivity and physiological modifications, producing a prediabetic state (92). This indicates that sleep is closely tied to the regulation of glucose metabolism. Alterations in sleep patterns increase the risk of developing pathologies related to body weight (77, 93).

## 5 The impact of sleep disorders on physical activity

Sleep is widely acknowledged to play an essential role in maintaining healthy mental and physical functioning (94, 95). Despite this significance, there is still a lack of understanding regarding the rationale for why people sleep. Sleep's fundamental function in metabolic balance has been established, and recent investigations have shown that sleep regulates key molecular pathways (96). Light, jet lag (97), and diet (98) are just a few of the environmental elements that can affect how long and how well you sleep, but genetics also plays a role (99). Despite the complexity surrounding the necessity, rationale, and results of sleep, sleep is an essential function for humans. Human adaptability to physiological and psychological stressors has a significant impact on physical activity outcomes and is influenced by a wide range of factors, such as prior experience, fitness level, level of motivation, and natural variations in physiological and behavioral processes across the 24-h cycle (100, 101). The SCN in the hypothalamus regulates these circadian cycles (102). Humans are extremely sensitive to changes in their natural environment, especially through the light–dark cycle, but the SCN cannot always maintain full control over these patterns (94).

Sleep is a recurrent, brief, and highly functioning state governed primarily by neurological mechanisms. Insomnia and other sleep disorders are increasingly identified in people of all ages. Sleep disorders are risk factors for depression, mental illness, heart disease (103), metabolic syndrome, and high blood pressure (104). Stress, anxiety, stimulant use, and exposure to electronic devices before bedtime are all known to diminish the quality of sleep (105). Increasing research suggests that diet, physical activity, and good sleeping habits can exert major influences on how well one sleeps (106). Tsunoda et al. reported that people who lead active lives sleep better and for longer than their sedentary counterparts. The quality of sleep can be improved without the use of pharmaceuticals if we increase our daily physical activity, outdoor exposure, and participation in activities such as walking (107). Moreover, in adults, regular exercise improves sleep quality when practiced over time. Increasing one's activity time and step count leads to better sleep, thus reinforcing the idea that any form of physical exercise is better than none (21). Jannsen et al. found in a recent review that infants with higher total physical activity levels tend to have shorter sleep durations and fewer naps during the day (108). Nevertheless, children and preschoolers with higher daily activity levels report better sleep quality and more consistent sleep patterns, suggesting that physical activity improves sleep (108). The quality of sleep is also influenced by the intensity of exercise. For example, preschoolers who engage in mild forms of physical activity tend to stay up later at night (109). Moreover, 73% out of 91 adolescents (11–19 years old) in Fairbrother et al.’s study reported problems staying asleep, and 65% had trouble falling asleep (110). Regular moderate-to-strong exercise is correlated with later bedtimes and shorter total sleep durations. In contrast, children between the ages of one and three who engage in more physical activity have improved sleep quality, fall asleep faster, and have fewer nighttime awakenings (108).

**Figure 1:**
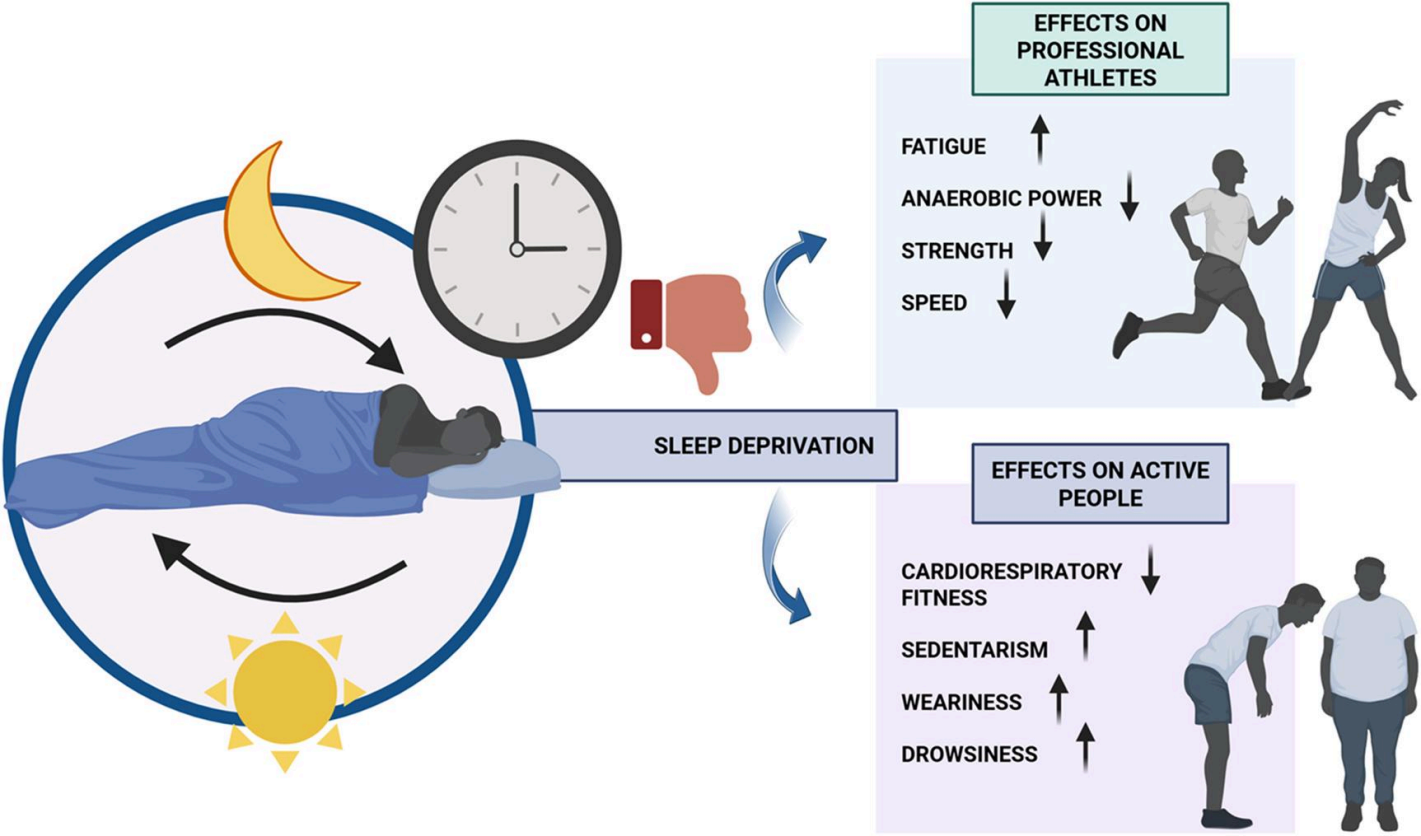
The effects of sleep deprivation on the athletic performance of both professional athletes and active individuals.

As already observed, physical exercise may affect our ability to sleep, but there is a reverse scenario where sleep disorders affect athletic performance and regular physical activity habits (Figure 1) (6). Endogenous circadian rhythms and typical sleep–wake cycles can become desynchronized. When individuals go to bed later than usual or get up earlier than usual, this is termed sleep restriction (SR). On the other hand, SD is the result of chronic sleep loss in which a person obtains little or no sleep for an extended period (i.e., many nights) (111). Findings by Mougin et al. indicate that SR increases physiological demand during athletic performances, leading to fatigue in athletes at a faster rate than normal (112). It appears that submaximal strength, muscular power (40), and anaerobic power all decrease after SR (45, 113). While these results suggest that SR hinders several aspects of sports performance, it remains unclear if sleep is crucial to performance in all athletes who experience brief, one-time spells of SR. However, poor sleep quality and quantity can trigger an imbalance in the autonomic nervous system (ANS), mimicking the overtraining condition. Sleep deprivation has been linked to an increase in proinflammatory cytokines, which may contribute to immune system malfunction. Furthermore, many studies have found that cognitive functioning slows down and becomes less accurate when people do n't get sufficient sleep (6). In addition, although sufficient data suggest that SD can have a major effect on aspects of athletic performance (54), the exact effect of SD on exercise performance is unclear (114, 115). This is especially relevant for the amount of time it takes to become completely exhausted while running for longer than 30 min (116). However, Symons et al. found no difference between SD participants and a control group on several maximum isometric and isokinetic strength tests for the upper and lower body after 60 h. Several studies have confirmed that one's grip strength performance does not decline with time spent awake (114). Nevertheless, Skein et al. noted that after 30 h of SD, 10 team sport athletes experienced slower mean sprint speeds, decreased muscle glycogen concentration, lower voluntary force and activation during maximal isometric knee extensions, and an increase in perceptual effort (54).

Both SD and SR may arise due to many factors in athletic contexts, such as travel schedules or training/playing at night (117). Sleep disruptions can increase homeostatic pressure, as well as have effects on emotional control, core temperature, and circulating melatonin, thereby delaying the onset of sleep. After many such occurrences, sleep deprivation and impaired cognitive and physiological functioning may set in (118). Erlacher et al. reported that while many athletes claimed that sleep disruption had little effect on their performance, others noted negative side effects such as a bad disposition the following day (119). Furthermore, 15% of the athletes reported experiencing nightmares in the 12 months prior to a major competition or game (120). Lastella et al. found that approximately 70% out of 103 athletes experienced poorer sleep than usual on the night before a competition, falling well below the recommended 8 h of sleep for healthy adults. Common reasons included nerves, noise, toilet breaks, and early start times (121). However, some studies reported that there was no significant shift in heart rate variability or other measures of autonomic nervous system activity among top female athletes in team sports just before a big game (122). In contrast, Edmonds et al. observed reduced parasympathetic and/or primary sympathetic modulation in athletes prior to a game, with their autonomic responses to standing dramatically reduced during and after the game for up to 4 days (123).

Although sleep is widely acknowledged to be important for human and athletic performance, the literature remains divided on whether or not a lack of sleep leads to a reduction in actual, measurable performance (124). Athletes may experience sleep deprivation right before a competition or if they are required to exercise at unusually early hours, but there is less proof of this happening in team sports (125). Contradictory evidence exists for the effect of acute SR. Performance during maximal one-off efforts (in particular for maximal strength) is generally maintained, but SD appears to have a negative impact on exercise performance (particularly endurance and repeated exercise bouts) (6).

## 6 The impact of sleep duration and quality on physical activity performance and recovery

Sleep is a fundamental human behavior that plays a crucial role in biopsychosocial development and has significant implications for biological, physical, psychological, and cognitive health (126). Although research in this area remains limited, empirical studies focusing on sleep in professional athletes have increased in recent years. Sleep health has a profound impact on various dimensions of professional athletics, including training, injury risk, recovery, and actual performance.

### 6.1 The impact of sleep on performance

The role of sleep in supporting training efforts is crucial, as it not only facilitates adherence to demanding training schedules but also maximizes the benefits derived from training by enabling peak effort and endurance. Several studies have specifically examined the impact of sleep on the training of professional athletes (127–130). For instance, a study by Peacock et al. (127) explored the training camp of professional mixed martial arts athletes over a 6-week period, finding that better sleep quality and regularity were associated with fewer missed practice sessions, likely due to reduced fatigue, illness, and injuries. Similarly, Fitzgerald et al. (128) demonstrated that reduced sleep quantity was linked to a higher incidence of illness among professional Australian football athletes. Moreover, Peacock et al. revealed that improved sleep characteristics, such as reduced sleep latency, were correlated with enhanced physical performance during the 6-week training period (127). Teece et al. (130) highlighted the negative effects of short sleep duration on the aerobic capacity of professional rugby athletes during preseason training. In addition, Serpell et al. (129) identified significant relationships between sleep duration and efficiency and salivary testosterone and cortisol levels in a sample of professional rugby athletes, highlighting the impact of these hormones on training ability and outcomes.

Furthermore, recent reviews are suggesting a strong positive correlation between sleep and athletic performance, including sports-specific skill execution, strength, and anaerobic power (131). It is a fundamental requirement for overall health and recovery, facilitating homeostatic processes that rejuvenate and replenish major physiological and psychological functions of the body (132). The optimal duration of sleep for athletes remains a topic of debate, with recent studies suggesting that healthy adults should aim for 7–9 h of sleep per night to support daytime functioning (133). Athletes are advised to prioritize approximately 8 h of sleep per night to prevent the neurobehavioral deficits associated with sleep deprivation (132).

Insufficient sleep has been shown to negatively affect both physiological and psychological performance (134). The primary psychological impact of sleep deprivation is altered mood states, impaired decision-making skills, and cognitive impairment (135). Decision-making skills are crucial in sports, and when sleep duration and quality are compromised, the cognitive processes involved in decision-making during sports are impaired, leading to decreased performance outcomes (117). While the physiological effects of sleep loss are less pronounced, they include reduced immune function (due to decreases in natural killer T cells) (117), decreased submaximal sustained performance (134), and even reduced glucose metabolism, which can contribute to increased fatigue.

Various factors, such as gender and the type of sport or exercise, can influence an athlete's sleep patterns. Some studies suggest that women generally have better sleep quality compared to men of the same age range. However, others argue that the effects of sleep deprivation are similar for both men and women (135). Different sports also impact athletes' sleep patterns, with factors such as training volume, intensity, frequency, psychological stress (especially during precompetition training), and external factors, including work obligations, family relationships, and academic commitments, all playing a role (136).

These findings highlight that sleep is a fundamental human behavior that plays a crucial role in biopsychosocial development and has significant implications for biological, physical, psychological, and cognitive health. While the research in this area is still limited, there has been an increase in empirical studies focusing on sleep in professional athletes. Sleep health has a profound impact on various dimensions of professional athletics, encompassing training, injury risk, recovery, and actual performance.

### 6.2 Sleep duration and physical activity performance

Sufficient sleep duration has been consistently associated with improved physical activity performance. Research suggests that individuals who obtain an adequate amount of sleep exhibit enhanced speed, power, accuracy, and reaction time during exercise. Longer sleep durations have been linked to increased endurance, improved muscular strength, and better overall athletic performance (137). Adequate sleep duration enables the body to replenish energy stores, repair damaged tissues, and optimize neural and physiological processes necessary for optimal physical performance (138). On the contrary, inadequate sleep duration has been shown to impair physical activity performance (139). Sleep deprivation negatively affects cognitive function, reaction time, motor coordination, and decision-making abilities, leading to decreased accuracy and reduced overall performance. Athletes who consistently experience insufficient sleep may experience fatigue, reduced motivation, and an increased risk of injuries during training or competition (140).

### 6.3 Sleep quality and physical activity performance

In addition to sleep duration, the quality of sleep also plays a vital role in physical activity performance. The stages of sleep, particularly the deep sleep stages (slow-wave sleep), are crucial for cellular repair, muscle recovery, and the release of growth hormone (60). During deep sleep, tissue repair and muscle protein synthesis occur, contributing to optimal recovery and adaptation from exercise-induced stress. Research indicates that poor sleep quality—characterized by frequent awakenings, disruptions, or inadequate time spent in the deep sleep stages—can negatively impact physical activity performance (141). Impaired sleep quality has been associated with decreased reaction time, decreased accuracy, diminished focus, and increased perception of effort during exercise. It can also lead to increased levels of inflammation, which may hinder recovery processes and increase the risk of overtraining syndrome.

### 6.4 Sleep duration and quality in recovery

Sleep serves as a critical period for recovery and adaptation after physical activity. During sleep, the body repairs damaged tissues, synthesizes proteins, restores energy reserves, and enhances immune function. Sufficient sleep duration and high-quality sleep are necessary for optimal recovery from exercise-induced fatigue and muscle damage (142). Inadequate sleep duration and poor sleep quality can prolong recovery time and compromise the body's ability to repair and adapt. Insufficient sleep restricts the release of growth hormone, which is essential for tissue repair and muscle growth (143). It also impairs glycogen restoration, negatively affecting energy replenishment for subsequent training sessions. Without adequate recovery, athletes may experience reduced performance, increased risk of injury, and heightened susceptibility to illness.

### 6.5 Sleep perturbances due to travel and time zone change

When athletes travel across time zones, their sleep health often deteriorates due to factors such as disruptions to their circadian rhythm, travel fatigue, and irregular sleep patterns (144). Roy and Forest (145) investigated the effects of time zone change on the performance of teams in the National Basketball Association (NBA), National Hockey League (NHL), and National Football League (NFL) over a five-season period (2010–2015) by analyzing winning percentages based on the direction of travel and game time. Their findings showed that traveling westward, but not eastward, was associated with a significantly lower winning percentage in the NBA and NHL, with a similar trend observed in the NBA. Interestingly, this effect was primarily observed in evening games, as no significant circadian disadvantage or advantage was found in afternoon games. The study also revealed a linear reduction in winning percentage for each additional time zone traveled, which was statistically significant across all three sports. Charest et al. (146) obtained similar results in their examination of the effects of travel on back-to-back games in the NBA, spanning multiple seasons. They found a 3.69% lower winning percentage when teams traveled westward compared to eastward. However, this study also highlighted the influence of factors beyond travel direction and time zone change—such as the sequence of games and distance traveled—on team performance. Glinski and Chandy (147) further supported the detrimental effects of westward time zone change on NBA performance in their study on the impact of jet lag on free throw shooting. Their results showed that teams had a significantly lower free throw percentage when experiencing jet lag (traveling across at least three time zones) compared to non-jet lagged games, but this effect was specific to westward travel and not observed in eastward travel. However, not all studies consistently support the notion that time zone change negatively affects performance in westward travel. Leota et al. (148) found that time zone changes in the eastward direction, but not westward, were associated with reduced NBA winning percentages and overall point, rebound, and field goal percentage differentials. Similarly, Zacharko et al. (149) concluded that eastward travel across time zones led to worse performance results compared to westward travel in professional soccer players. Lastly, McHill and Chinoy (150) reported lower winning percentages and decreased shooting accuracy, effort, and defensive performance when traveling across time zones, regardless of travel direction. However, the magnitude of these effects varied depending on the direction of travel. Overall, these findings suggest that time zone change, in any direction, is likely to negatively impact competitive performance, with sleep health being a central factor in these relationships (137).

## 7 The potential mechanisms underlying the relationship between sleep and physical activity, including the role of circadian rhythms, hormonal regulation, and neural pathways

The mechanisms regulating sleep have an impact on the gene expression of the central nervous system (CNS) and involve the endocrine, immune, and energy regulation systems. Sleep is determined by the activation of the SCN, which integrates information from all parts of the organism (151). This suprachiasmatic nucleus–hypothalamus axis regulates the sleep–wake cycle at the molecular level through a constant maintenance of energy by circadian fluctuations of enzymes involved in tissue metabolism (152). It is known that the circadian rhythm of sleep is generated through different feedback processes that are determined by the amount of ambient light on circadian genes within SCN cells (153, 154).

All information is integrated in the nuclei of the hypothalamus, allowing interaction between the anterior and posterior hypothalamus, the brainstem, and the basal forebrain, where the most essential neuromodulators in mammals are located, and which project to a large part of the olfactory system and the cerebral cortex (155). REM–NREM sleep cycles are regulated by the ultradian oscillator of the mesopontine junction and the transition between these cycles is modulated by the locus ceruleus and the nucleus oralis pontis reticularis (156). During non–rapid eye movement sleep (NREM) sleep, a decrease in sympathetic activity is evident, reducing overall activity of the organism. In contrast, REM sleep is associated with neuronal repair actions, including memory consolidation, synaptic homeostasis, brain maturation, and learning (157).

All this is also consistent with other mechanisms that are coordinated by the light–dark cycle, such as thermogenesis, glucose metabolism, intake, and lipid levels, which present modulations due to alterations in this circadian pattern (158). Therefore, when the quality and quantity of sleep are not adequate, different organic pathologies appear, such as alterations in insulin sensitivity and glucose tolerance, which are risk factors for developing diabetes, obesity, metabolic syndrome, and other diseases (159, 160).

Sleep has been directly linked to physical activity. The study of this field has seen increased interest in recent years, given the evidence of similar sleep patterns in patients with pathologies such as obesity, diabetes, or insulin resistance (161). Recent studies have shown that alterations in sleep patterns increase the expression of the antioxidant enzymes Glyoxalase-1 and Glutathione reductase, while increasing oxidative stress in the hippocampus, amygdala, and cerebral cortex (162). Moreover, physical activity and sleep are governed by distinct yet interconnected homeostatic physiological mechanisms that interact to facilitate sleep during periods of reduced light exposure. These mechanisms activate the somnogenic regions in the brain by lowering body temperature to prepare the organism for sleep induction (104).

Physical activity is closely associated with the regulation of ultradian cycles, whose activity decreases in the early afternoon. Physical exercise should be performed at a time that favors endogenous rhythms, considering the type of activity to be performed and individual factors, but ideally 4–5 h before going to sleep (163). Electroencephalogram (EEG) recordings show that individuals who perform regular physical activity exhibit slow brain wave activity across all sleep cycles. On the contrary, people with low or no physical activity presented less slow brain wave activity in addition to a greater predisposition to present depressive symptomatology. This indicates that these processes are interrelated and that these two systems work together to maintain organic homeostasis (164–166).

Sleep is also related to hormonal regulation. In relation to the immune system, proinflammatory cytokines are in charge of modulating sleep and their activity is altered with sleep deprivation (69, 167). Modifications of sleep–wake patterns have been observed in conditions such as fibromyalgia, major depressive disorder, or infectious diseases, directly impacting the immune system. Sleep is associated with the expression of T cells and the proinflammatory cytokines such as TNF-α and IL-6, which rise in the presence of sleep disturbances (168). In addition, the expression of cell adhesion molecules Mac-1 and L-selectin on lymphocytes and monocytes shows alterations in the circadian rhythm and its periodicity, linked to the modulation of leukocyte-induced pathogenesis of asthma or cardiovascular crises and strokes (169).

Different sleep cycles have an impact on glucose and insulin secretion, with hypoglycemia sometimes occurring at night. Glucose metabolism intensifies its activity through the influence of the SCN, which prepared the body for awakening and increased activity (170). Maintaining the circadian rhythm supports the proper circulation of afferent and efferent signals and protects against ANS dysfunctions such as diabetes or metabolic syndrome (171). Low melatonin levels promote insulin sensitivity, reduce blood pressure during sleep, and flattened melatonin rhythms (172).

Sleep dysfunction induces an increase in cortisol levels in the late afternoon, leading to very high amounts of glucocorticoids (101). During the physiological processes underlying the initiation of slow-wave brain activity, cortisol tends to be inhibited, and during this sleep period, orexins regulate food metabolism and energy balance as well as reward mechanisms, so that sleep restriction for one night is sufficient to affect leptin levels the next morning as well as cortisol rhythms (173).

In relation to neuronal pathways, the connections between these specialized cells are maintained during the sleep cycle. During this period, they assume the function of maintenance and repair, which allows the elimination of toxins that are stored throughout the day (174). As the day comes to an end, chemical signals moderate neuronal activity, inducing sleep (175). GABA or *γ*-aminobutyric acid is a neurotransmitter that acts as the main inhibitor of the CNS; in addition to other functions, and once it is activated, it blocks the transmission of information from motor neurons, favoring the reduction of excitation (176). On the other hand, norepinephrine and orexin are responsible for maintaining neuronal activity in certain regions of the brain during wakefulness. Other neurotransmitters such as acetylcholine, adrenaline, serotonin, and histamine are also present (177, 178).

The brainstem and the ascending reticular activating system are essential for cortex stimulation, particularly in the region of the posterior hypothalamus since this is where the waking state is maintained (179). With the release of glutamate, the cortex is activated via the dorsal pathway and the ventral pathway of the hypothalamus, projecting to the cerebral cortex and hippocampus (180). During sleep, the cerebral cortex and the thalamus interact. There are two neuronal circuits in the brainstem that act alternatingly to induce sleep or wakefulness; they are composed of reduced neuronal groups that form very complex circuits in a structural network to maintain homeostasis (181).

There are other brain regions that are involved in sleep modulation. According to recent studies, the area known as the pons sends signals to the thalamic nuclei and the cortex and communicates with the spinal cord to temporarily block motor activity (182). In addition to the reticular activating system, researchers have investigated whether the nucleus of the solitary tract, which is in the dorsal medulla, could create a link between the sleep–wake state and cardiovascular, respiratory, and gastrointestinal functions (183). Although it has no direct projection to the cortex, it does project to areas of the brainstem, thalamus, and hypothalamus, thereby reaching the cortex by innervation and modulating waves during sleep (184).

## 8 The impact of nutrition on sleep and physical activity

Individual differences in digestive and metabolic processes account for a large portion of the observed variability in dietary variables. In addition, hormones and inflammatory states can be directly or indirectly affected by nutrition, both of which contribute to sleeplessness. Although the role of diet in controlling sleep is multifaceted (185), several possible explanations have been proposed. To begin, certain parts of one's diet can have an immediate effect on one's ability to rest (186). In addition, the inflammatory status, which is also closely associated with sleeplessness, may be changed by long-term dietary changes (187). Numerous studies have demonstrated that sleep disruption is linked to increased levels of glucocorticoid and decreased levels of inflammatory cytokines (particularly C-reactive protein and interleukin 6) (104). Melatonin is a well-known sleep aid that informs the body about when it should be dark and when it should be light. The effects of melatonin on sleep and circadian rhythm are mediated by two G-protein-coupled receptors, MT1 and MT2. Thus, melatonin-containing foods have an effect on sleep quality (188).

Within this broader lifestyle context, recent evidence has emphasized the integrative role of chronotype and physical activity in shaping sleep and dietary behaviors. Evening chronotypes appear more vulnerable to poor sleep quality, whereas shifts toward a morning chronotype are associated with marked improvements in sleep quality alongside distinct dietary patterns, underscoring the interconnected nature of sleep, physical activity, chronotype, and nutrition (189).

### 8.1 Nutrition and sleep quality

**Figure 2:**
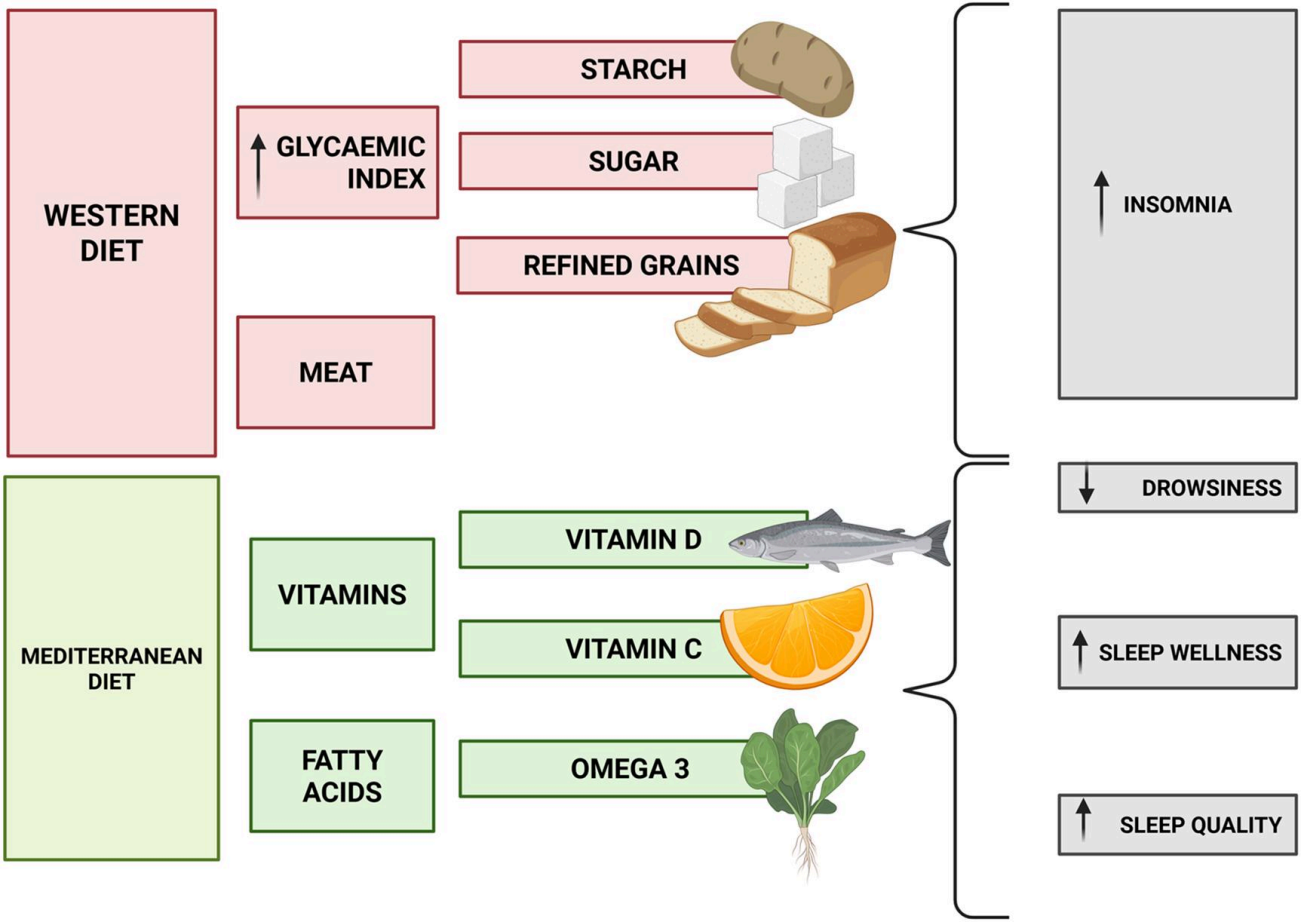
Dietary patterns affect sleep. The Western diet generates more insomnia than the Mediterranean diet. Vitamins D and C and omega-3 have been found to improve sleep health.

There has been a notable surge in research on the connection between dietary patterns and sleep quality over the past few decades. Studies have included therapeutic interventions and enhanced nutrition, as well as general information about the role of diet in sleep. A high-glycemic index (GI) diet has been linked to an increased risk of stroke, cancer, and other long-term illnesses (190). Rapid elevation of blood glucose after eating a high-GI diet prompts a counteracting rise in insulin production and subsequent humoral effects (191). Katigiri et al. reported inconsistent effects of carbohydrates on slumber. A high-GI diet was associated with increased incidence of insomnia over 3 years in their prospective study of a large population of postmenopausal women. In addition, higher intakes of added sugars, starch, and non-whole/refined grains were each associated with higher incidence of insomnia (Figure 2) (192). A study comparing the effects of a very low-carbohydrate diet consumed for 48 h increased the proportion of SWS and decreased the proportion of REM sleep (the “dreaming” stage of sleep) (193). However, carbohydrates are often recommended for better sleep because they boost tryptophan uptake by the brain, where it is metabolized into serotonin and melatonin (194) Nevertheless, not everyone will experience a consistent improvement in sleep quality after ingesting tryptophan because of the brain's conversion of tryptophan to serotonin, a neurotransmitter known to influence many distinct aspects of sleep and wakefulness (194, 195). Some serotonin is converted to melatonin, but the exogenous dose of melatonin required to affect sleep cannot be credibly supplied by diet (194). Saturated fatty acids appear to be detrimental to sleep health. Lengthy periods of ingestion of saturated fatty acids contribute to diabetes, which is frequently associated with sleep disorders (196). However, according to research, a diet low in omega-3 polyunsaturated fatty acids (PUFAs) disrupts nocturnal sleep by influencing the melatonin rhythm and circadian clock functioning (197). In obese patients with obstructive sleep apnea syndrome, there is also a beneficial relationship between omega-3 fatty acid composition in gluteal adipose tissue and sleep wellness, including slow-wave sleep and rapid-eye movement sleep (198).

Gao et al. reported that a lack of vitamin D is linked to an increased risk of sleep disorders such as poor sleep quality, short sleep duration, and drowsiness (199). When individual studies were examined, most of them revealed a beneficial relationship between vitamin D intake and sleep quality (199). Cross-sectional research of people in the United Kingdom found a link between fruit and vegetable consumption and sleep wellness, with long sleepers showing high plasma vitamin C levels (200). The benefits of vitamin B12 on sleep are likewise debatable. A case study revealed that vitamin B12 treatment was effective in treating a free-running sleep–wake cycle and delayed sleep phase syndrome (201). However, sleep length has also been connected to micronutrient status, with sleep duration being positively associated with Fe, Zn, and Mg levels but adversely associated with Cu, K, and vitamin B12 levels (202). Overall, however, the findings support that micronutrient-rich diets, such as plant-based diets, are positively associated with improved overall sleep health (203, 204). Moreover, Scoditti et al. showed that higher adherence to a Mediterranean diet is connected with adequate sleep duration and numerous indications of better sleep quality (205). An interesting study carried out by Jansen et al. reported that a healthy plant-based diet and a breakfast-type food pattern were linked to earlier sleep time 2 years later. It has also been suggested that a plant-based diet was linked to shorter sleep phase delays over time. A meat- and carbohydrate-based diet pattern, on the other hand, was linked to more social jet lag. These findings suggest the potential benefits of a nutritious diet, in addition to other sleep hygiene principles, may help teenagers in achieving and maintaining adequate sleep (206).

### 8.2 Nutritional interventions and how they affect sleep quality and performance

**Figure 3:**
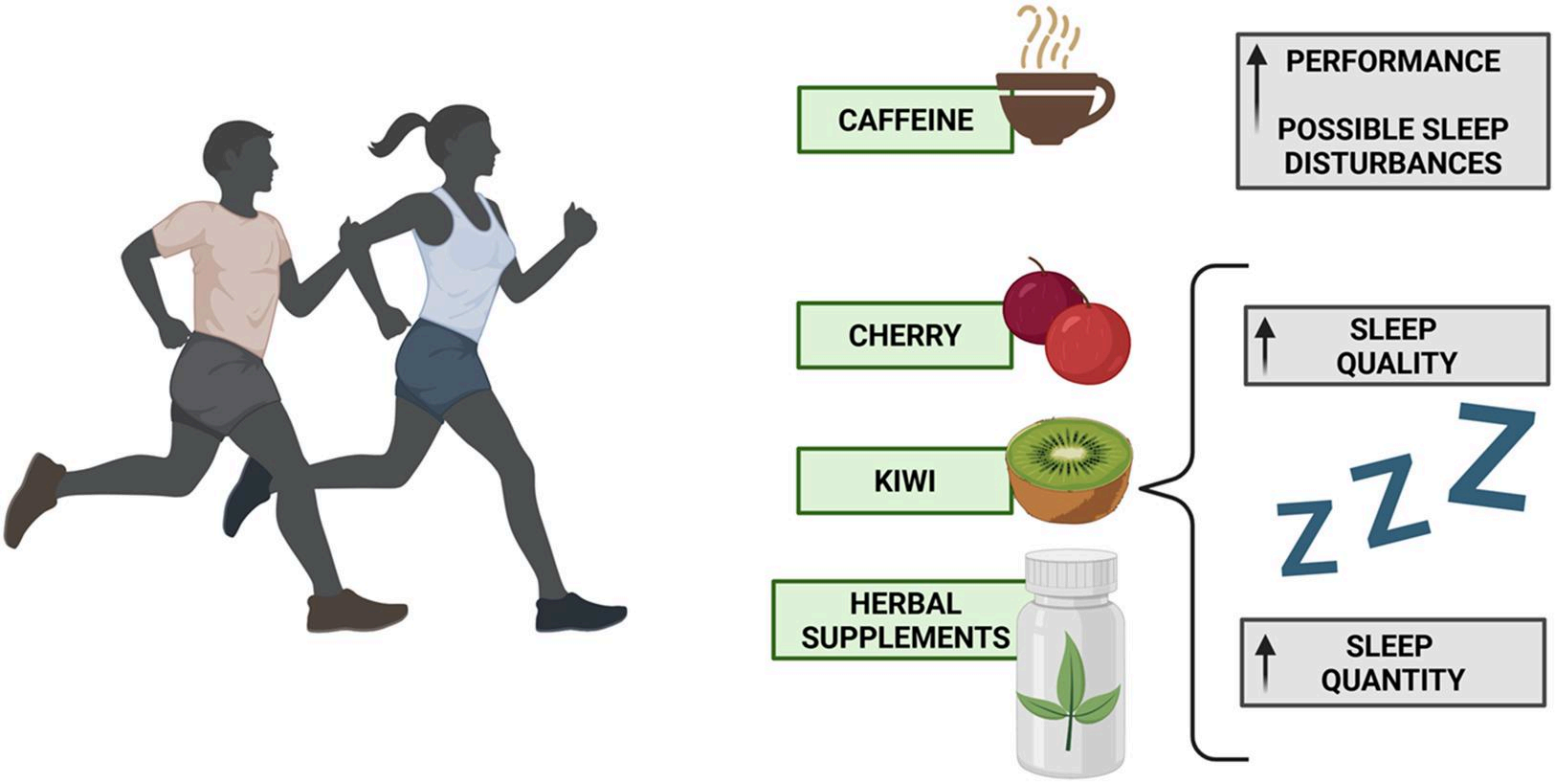
Diet management in athletes will influence their performance as well as their sleep health. Herbal supplements, cherry, or kiwi will improve sleep, while caffeine can worsen sleep and improve performance.

Diet has been demonstrated to significantly affect sleep in general populations; however, less is known about its effects on sleep in athletically trained groups. Barnard et al. reported that evening consumption of high-glycemic index carbohydrates and protein rich in tryptophan may reduce sleep latency. Although promising, more research is required to clarify the impact of probiotics, cherry juice, and beetroot juice on the sleep of athletes. Athletes experiencing sleep difficulties should be screened for caffeine use and trial dietary strategies (e.g., evening consumption of high-GI carbohydrates) to improve sleep (207). Gratwicke et al. suggested that the ingestion of carbohydrates to enhance sleep parameters is inconclusive, though food with a high glycemic index appears to offer modest benefits. It has been shown that tart cherry juice improves sleep quantity, herbal supplements improve sleep quality, and kiwifruit and protein interventions improve both (Figure 3) (208). However, other studies have supported the idea that daily energy intake is negatively correlated with productivity and nocturnal total sleep time, and positively correlated with wake after sleep onset (WASO), regardless of the nocturnal GI meals (209). Vlahoyiannis et al. indicated that eating a high-glycemic index lunch after a single session of spring interval training can increase sleep efficiency and decrease sleep onset latency. The enhancements in sleep had no negative effects on the subjects' capacity to leap or their aerobic endurance. On the other hand, better sleep was correlated with faster reaction times in visual tasks (210). Nevertheless, caffeine supplementation (6 mgkg1) did not enhance the 800-m running performance of trained runners but did affect their sleep quality (211). On the contrary, Miller et al. specified that caffeine can improve athletic performance; however, athletes should be wary of the stimulant's negative impact on sleep if they consume it regularly (particularly for late afternoon/evening training and competition) (212). Overall, the scientific literature suggests that athletes and sports professionals can improve sleep and performance by intentionally manipulating macronutrient timing and type, dietary supplementation, and caffeine intake.

## 9 The potential synergies between physical activity, sleep, and nutrition interventions

The potential synergies between physical activity, sleep, and nutrition interventions have garnered increasing attention in recent years, with numerous studies exploring the interconnectedness of these lifestyle factors. The interplay between physical activity and sleep has been investigated in several studies. For instance, Chaput et al. (213) conducted a study involving adults and found that higher physical activity levels were associated with improved sleep duration and quality. These findings are supported by Sanz-Martín et al. (214), who reported a positive correlation between physical activity and sleep duration among adolescents. Similarly, it has been demonstrated that regular physical activity was linked to decreased sleep latency and increased sleep efficiency. These studies collectively suggest that engaging in physical activity may positively influence sleep patterns. Moreover, the timing of exercise in relation to sleep has been explored. Youngstedt et al. (5) found that engaging in moderate-intensity aerobic exercise in the morning led to improved sleep quality. In contrast, evening exercise was associated with prolonged sleep latency (5). These findings highlight the importance of considering exercise timing when examining the relationship between physical activity and sleep.

In terms of nutrition and sleep, the influence of macronutrient composition on sleep quality has been investigated. Afaghi et al. (215) observed that a higher carbohydrate intake was associated with improved sleep quality and decreased sleep onset latency. Similarly, St-Onge et al. (167) reported a positive correlation between carbohydrate consumption and sleep efficiency (216). In addition, protein intake has been implicated in sleep outcomes. Higher protein intake has been linked to fewer awakenings during sleep (215). These findings suggest that dietary choices, particularly the consumption of carbohydrates and protein, may impact sleep quality. Micronutrient deficiencies have also been associated with sleep disorders. Grandner et al. (217) highlighted that deficiencies in iron, magnesium, and vitamin D were linked to an increased risk of sleep disorders. Similarly, Gao et al. (199) reported an association between low vitamin D levels and poor sleep quality. Ensuring adequate intake of these micronutrients through a balanced diet or supplementation may potentially improve sleep quality.

Integrating interventions targeting physical activity, sleep, and nutrition may yield synergistic effects on various health outcomes. Mikkelsen et al. (218) conducted a study that examined the effects of a combined intervention on mental health. They found that a 12-week combined intervention significantly reduced symptoms of depression and anxiety compared to single-component interventions. This suggests that addressing multiple lifestyle factors simultaneously may yield greater mental health benefits. Furthermore, combined lifestyle interventions encompassing physical activity, sleep, and nutrition components have shown promise in improving metabolic health and reducing chronic disease risk factors. For example, it was reported that a combined lifestyle intervention led to greater improvements in insulin sensitivity and lipid profiles compared to individual interventions targeting only one lifestyle factor (219).

## 10 Wearable technology for monitoring sleep and physical activity

Sleep assessment has gained significant attention in recent decades, leading to the development and incorporation of various devices to evaluate sleep quality. PSG is widely recognized as the gold standard for assessing sleep since its introduction in clinical practice in 1974 (220). PSG is a comprehensive technique that involves the simultaneous monitoring of multiple dimensions of sleep. Typically, a PSG recording includes measurements of central, frontal, and occipital EEG activity, eye movements, chin and other muscle activity (EMG), electrocardiogram, pulse oximetry, respiratory efforts, nasal and oral airflow, and body position (220). Depending on individual needs and conditions, PSG can be complemented with additional sensors, allowing for a more detailed evaluation of sleep patterns and characteristics (221).

Although PSG provides the most accurate and comprehensive assessment of sleep, it is a complex, expensive, and time-consuming procedure (222). It requires trained technicians to set up the equipment and specialized expertise to analyze the collected data. Consequently, self-reported sleep measures have been widely used as a more accessible alternative for assessing sleep quality. Sleep diaries and questionnaires, for instance, offer subjective estimations of various sleep-related parameters and are commonly employed for initial screening of sleep disorders (7). Several questionnaires have been developed specifically for sleep assessment, including the Karolinska Sleep Diary (223), the Pittsburgh Sleep Quality Index (PSQI) (224), the Epworth Sleepiness Scale (225), and the Sleep Hygiene Index (226), as well as questionnaires tailored to athletes such as the Athlete Sleep Behavior Questionnaire (227) and the Athlete Sleep Screening Questionnaire REF (228). These instruments provide valuable insights into subjective sleep experiences and can serve as effective screening tools for identifying potential sleep issues.

Another objective method for assessing sleep/wake behavior, particularly in certain populations, is actigraphy. Actigraphy has gained popularity as a reliable alternative to PSG. Actigraphic devices typically include triaxial (3D) or biaxial (2D) accelerometers that record movement patterns over time. Through specific algorithms, these devices estimate sleep/wake behaviors based on activity levels. Actigraphy indirectly measures sleep by analyzing individuals' motor activity levels, assuming that fewer movements occur during sleep (134). Various sleep parameters—such as total sleep time, SOL, SE, and WASO—can be derived from the wakefulness/sleep data collected by actigraphy (134). Actigraphy offers a convenient and practical method for objectively measuring sleep in both athletes and non-athletes. It is more affordable than PSG, requires less expertise in device setup and data analysis, and allows for long-term sleep monitoring. Actigraphy can be performed in participants' habitual sleep environments, such as their own homes and bedrooms, with minimal discomfort.

The market for consumer wearable devices has experienced remarkable growth, providing individuals with the means to monitor their physical activity, sleep, and other behaviors (229). The growing interest in sleep among the general population has been facilitated by the introduction of commercial wearables and nearables designed for sleep tracking. Wearable technology refers to electronic devices and systems integrated into clothing or worn on the body (230). Smartwatches, GPS-enabled sneakers, and wristbands are examples of wearable devices that have become increasingly popular. These devices offer a combination of user-friendly features and affordability, allowing for simple and long-term sleep monitoring. They do not require expertise as most of them do not export raw data but utilize proprietary algorithms to analyze and interpret the collected data. These devices are typically connected to remote applications that directly display the results (229). Dedicated sleep trackers have emerged as a niche within the wearable market. These devices focus solely on monitoring sleep-related metrics and are designed to be worn during the night. They employ a combination of sensors—such as accelerometers, gyroscopes, and sometimes even ambient light sensors—to capture detailed information about sleep duration, sleep stages, and sleep interruptions. Some sleep trackers also offer features like snore detection and analysis of sleep positions. Moreover, wearable devices in the form of rings have also entered the sleep tracking market. These rings are worn on the finger and utilize various sensors, such as pulse wave sensors, to gather data on sleep patterns. The compact design of these rings allows for unobtrusive and comfortable sleep monitoring. To analyze and interpret the data collected by wearable devices, manufacturers typically provide companion mobile applications or web platforms. These applications display sleep metrics, generate sleep reports, and offer insights into sleep patterns over time. Many of these platforms leverage machine learning algorithms to provide personalized recommendations for improving sleep quality.

## 11 Personalized interventions for optimizing sleep and physical activity habits

In recent years, there has been a growing interest in developing personalized interventions to optimize sleep and physical activity habits. Personalization is crucial because individuals vary in their sleep patterns, activity levels, preferences, and behaviors. By tailoring interventions to meet the specific needs and characteristics of individuals, it is possible to enhance the effectiveness and engagement of interventions aimed at improving sleep and physical activity. Research has explored the use of personalized interventions in the context of sleep and physical activity. Ghanvatkar et al. (231) conducted a scoping review on personalized physical activity interventions, highlighting the importance of considering individual variations in activity levels, requirements, preferences, and behavior when designing interventions. Personalized approaches were shown to promote and sustain physical activity behaviors. Furthermore, a study by Shimamoto et al. (232) investigated the effectiveness of brief personalized interventions for improving physical activity and sleep among older adults. The study utilized mHealth strategies—including self-monitoring, motivational messages, activity reminders, and phone coaching—to facilitate participation and engagement in physical activity. The findings suggested that personalized interventions delivered through mobile health platforms have the potential to positively impact both physical activity and sleep behaviors.

Recent research has demonstrated the effectiveness of personalized interventions in improving sleep outcomes and physical activity levels. For example, a recent systematic review showed how physical activity programs have positive effects on sleep outcomes, particularly in the adolescent population (233). Another study by Ghanvatkar et al. (231) highlighted the importance of personalization in mobile health interventions aimed at increasing physical activity. These findings emphasize the need for tailored approaches to address sleep and physical activity habits. Moreover, combining interventions targeting both physical activity and sleep quality has shown promise in improving overall health outcomes. In another study, the authors implemented a mobile health combined behavior intervention focusing on physical activity and sleep quality, which resulted in positive changes in participants' behaviors (234). This suggests that personalized interventions addressing both domains can lead to more comprehensive and effective outcomes.

A randomized controlled pilot trial investigated the preliminary effect of a 24-week mHealth-facilitated personalized intervention on physical activity and sleep in community-dwelling older adults. The intervention included personalized exercise prescription, training, goal setting, and financial incentives, with the incorporation of mHealth strategies such as self-monitoring, motivational messages, activity reminders, and phone coaching. The study found positive outcomes in terms of improving physical activity levels and sleep quality among the participants (235). Another scoping review found that personalized goal setting and daily logging with dynamic feedback improved overall sleep quality, subjective sleep quality, sleep onset latency, wake-time variability, sleep hygiene, and insomnia severity (236).

In another study where authors reviewed the relationship between sleep and physical activity and their impact on human health, they proposed several physiological mechanisms between sleep and exercise, including the influence of physical activity on sleep quality and duration, as well as the effects of sleep on physical activity performance and recovery. There is also a bidirectional relationship between sleep and physical activity, suggesting that those with sleep disorders may benefit from engaging in increased physical activity (237). A systematic review of the interrelationship between sleep and exercise showed that higher leisure-time physical activity was correlated with better sleep, suggesting that engaging in leisurely physical activity can improve sleep quality. Conversely, the authors also suggested that the relationship between sleep disorders and physical activity is bidirectional, with sleep disorders resulting in greater fatigue throughout the day, thus lowering the likelihood of exercising (19). Another scoping review examined the effect of various physical activity intervention strategies on sleep across different populations. The review found that personalized goal setting and daily logging with dynamic feedback can improve overall sleep quality, subjective sleep quality, sleep onset latency, wake-time variability, sleep hygiene, and insomnia severity. Other intervention programs—including behavioral feedback, goal setting, or health coaching programs—provided behavioral support to improve adherence through phone contact, email connect, or group sessions (238).

Collectively, these studies provide valuable insights into the potential benefits of personalized interventions for optimizing sleep and physical activity habits. By considering individual characteristics and tailoring interventions accordingly, it is possible to enhance intervention outcomes and promote healthier sleep and activity behaviors.

## 12 Future lines of research

Future research in the field of sleep and physical activity should focus on several key areas to expand knowledge and provide comprehensive insights into this complex interplay. The following lines of research are suggested:

Longitudinal studies: Examine the bidirectional relationship between sleep and physical activity over extended periods. This will help establish causal relationships and determine how changes in one behavior influence the other over time.
Intervention studies: Investigate the effectiveness of different physical activity interventions in improving sleep quality and reducing the risk of sleep disorders. Explore various types and intensities of physical activity interventions and their specific effects on sleep outcomes.
Mechanistic studies: Further investigate the underlying mechanisms linking sleep and physical activity, including the role of circadian rhythms, hormonal regulation, and neural pathways. This will provide a deeper understanding of the physiological processes involved in the interplay between sleep and physical activity.
Sleep disorders and physical activity: Explore the impact of different sleep disorders on physical activity levels and patterns. Investigate how sleep disorders, such as insomnia or sleep apnea, affect physical activity engagement, performance, and adherence to exercise programs.
Individual differences: Investigate individual differences in the relationship between sleep and physical activity. Explore how factors such as age, sex, chronotype, and genetic variations influence the interplay between sleep and physical activity.
Technology and interventions: Explore the potential of wearable technology and personalized interventions for monitoring and enhancing sleep and physical activity habits. Investigate the effectiveness of mobile applications, wearable devices, and personalized feedback systems in promoting healthy sleep and physical activity behaviors.

## 13 Conclusion

This narrative review highlights the complex and dynamic bidirectional relationship between sleep and physical activity, emphasizing their joint relevance as modifiable lifestyle behaviors with profound implications for physical, metabolic, and mental health. The evidence consistently indicates that regular physical activity, particularly at moderate intensity, is associated with improvements in sleep quality, while adequate sleep duration and quality are essential for optimizing physical performance, recovery, and long-term engagement in physical activity. Importantly, these relationships are not linear but are shaped by multiple interacting factors, including exercise timing, intensity, chronotype, nutritional habits, and individual characteristics.

Despite a robust body of evidence supporting the beneficial effects of physical activity on sleep quality, important inconsistencies remain—particularly regarding sleep duration, high-intensity exercise, and evening training. Much of the current literature is observational, limiting causal inference, and intervention studies often focus on short-term outcomes in relatively homogeneous adult populations. Consequently, key gaps persist concerning population-specific responses, sex differences, long-term adherence, and interindividual variability in responsiveness to exercise-based sleep interventions.

This review also underscores the integrative role of nutrition and chronotype in shaping sleep–physical activity interactions, reinforcing the need to move beyond isolated behavioral approaches. Emerging evidence suggests that combined lifestyle interventions targeting sleep, physical activity, and dietary patterns may yield synergistic benefits for metabolic health, mental wellbeing, and disease prevention. In this context, advances in wearable technology and digital health tools offer promising opportunities to monitor sleep and physical activity objectively, support personalized interventions, and improve long-term adherence.

Future research should prioritize well-designed longitudinal and randomized controlled studies that incorporate objective measurements, diverse populations, and sufficient follow-up durations to clarify causal pathways and optimize intervention design. Embracing personalized, chronobiologically informed, and multisystem approaches will be essential to translating the growing body of evidence into effective, scalable strategies for improving sleep health, physical activity engagement, and overall wellbeing.
